# Crystal structures of Fsc1, a novel autophagy factor that mediates autophagosome–vacuole fusion in fission yeast

**DOI:** 10.1107/S205979832600197X

**Published:** 2026-03-25

**Authors:** Chidiogo Azuka, Jian Liu, Xiangshu Jin

**Affiliations:** a Department of Chemistry Michigan State University East Lansing MI48824 USA; University of Queensland, Australia

**Keywords:** Fsc1, fasciclin domain, autophagy, autophagosome–vacuole fusion

## Abstract

Fsc1 is a recently identified autophagy factor in the fission yeast *Schizosaccharomyces pombe* that is implicated in the autophagosome–vacuole fusion step during the final stages of autophagy. Despite its critical role, the structural basis of Fsc1 function has remained unknown. Here, we report the first crystal structures of the luminal domain of Fsc1, revealing an elongated, modular architecture composed of five tandem fasciclin (FAS1) domains. Each domain adopts a hallmark β-sandwich fold, and the overall assembly forms a continuous scaffold featuring a conserved surface groove within the FAS1-4 domain. Structural and biochemical analyses demonstrate that Fsc1 forms a homodimer in solution through a shared interface observed in two independent crystal forms, supporting a biologically relevant but potentially low-affinity association. Comparative sequence and structural analyses reveal significant homology between Fsc1 and human fasciclin proteins, including TGFBI and periostin, suggesting similar structural principles underlying their functions. Together, these findings provide the first structural insights into Fsc1 and establish a structural framework for understanding how its modular architecture and context-dependent dimerization may facilitate late-stage membrane fusion during autophagy.

## Abstract 2

Crystal structures of the autophagy protein Fsc1 reveal conserved fasciclin features and a stable dimer interface that illuminate its possible role as a scaffold in autophagosome–vacuole membrane fusion.

## 1. Introduction

Macroautophagy (hereafter autophagy) is a conserved eukaryotic degradation pathway that is crucial for cellular homeostasis and adaptation to stress conditions such as nutrient starvation (Xu & Du, 2022 ▸; Parzych & Klionsky, 2014 ▸). During autophagy, cytoplasmic components such as organelles and macromolecules are sequestered into double-membrane vesicles known as autophagosomes. These vesicles subsequently fuse with lysosomes (or vacuoles in yeast) for degradation by hydrolytic enzymes (Parzych & Klionsky, 2014 ▸; Yu *et al.*, 2020 ▸). Dysregulation of autophagy has been implicated in a range of pathologies, including neurodegenerative diseases, cancer and aging, underscoring the importance of understanding the molecular mechanisms governing this pathway (Parzych & Klionsky, 2014 ▸; Vargas *et al.*, 2023 ▸).

Autophagy is mediated by a set of proteins, collectively known as autophagy factors, and proceeds through three major stages: initiation, elongation and fusion. In the more extensively studied budding yeast *Saccharomyces cerevisiae*, initiation of autophagy begins at punctate sites within the cell known as the phagophore assembly site (PAS). At these PAS sites, the Atg1–Atg13–Atg17–Atg31–Atg29 kinase complex activates the autophagy pathway (Wen & Klionsky, 2016 ▸). The PtdIns3K complex, composed of Vps34–Vps30/Atg6–Vps15–Atg14–Atg38, is recruited next and drives membrane nucleation (Wen & Klionsky, 2016 ▸; Hu & Reggiori, 2022 ▸). Following nucleation, the phagophore expands to form the double-membraned autophagosome; this elongation process involves two conjugation systems. The first is formed by the Atg12–Atg5–Atg16 complex (via Atg7 and Atg10), and the other involves Atg8 conjugation to phosphatidylethanolamine through a series of processing steps by Atg4, Atg7 and Atg3 (Parzych & Klionsky, 2014 ▸; Wen & Klionsky, 2016 ▸; Hu & Reggiori, 2022 ▸). Additional factors, including Atg9, contribute to phagophore expansion through its interaction with the Atg2–Atg18 complex (Hu & Reggiori, 2022 ▸; Chumpen Ramirez *et al.*, 2023 ▸). The autophagosome matures and encloses cargo, assisted by the Rab5-like GTPase Vps21 and Atg14 (Zhou *et al.*, 2019 ▸). Upon maturation, the autophagosome fuses with the vacuole, a process typically mediated by HOPS complex and SNARE proteins, including Ykt6, Vam3, Vam7 and Vti1 (Hu & Reggiori, 2022 ▸; Shvarev *et al.*, 2022 ▸). Although the core mechanisms of this terminal fusion step are conserved, notable differences exist between budding yeast and fission yeast, with fission-yeast proteins exhibiting several features more closely resembling those of higher eukaryotes.

The fission yeast *Schizosaccharomyces pombe*, which is evolutionarily distant from *Saccharomyces cerevisiae*, shares several cellular features with humans, including gene-regulation mechanisms (Vyas *et al.*, 2021 ▸), and serves as a valuable model for autophagy studies (Zhao *et al.*, 2016 ▸). While numerous autophagy proteins are conserved across *S. cerevisiae*, *S. pombe* and mammals, several additional proteins, such as Atg101 and the Atg11/FIP200 homologs, are present in *S. pombe* and mammals but are absent in the budding yeast *S. cerevisiae* (Xu & Du, 2022 ▸). These differences highlight the limitations of the budding yeast *S. cerevisiae* as a universal model and underscore the utility of *S. pombe* for investigating conserved autophagy mechanisms relevant to human biology.

A genome-wide screen by Sun *et al.* (2013 ▸) identified a novel autophagy factor in *S. pombe*: Fsc1, a fasciclin (FAS1) domain-containing protein. Their study implicated Fsc1 in the poorly understood step of autophagosome–vacuole fusion during autophagy. Under starvation conditions autophagy is triggered, and Fsc1 localizes to the vacuolar rim, consistent with a role in mediating fusion through its localization to the vacuolar membrane (Sun *et al.*, 2013 ▸). Deletion of *fsc*1 impairs this key autophagic step, leading to the accumulation of mature autophagosomes within cells; this phenotype has been serendipitously leveraged for transmission electron microscopy (TEM) studies of autophagosome structure during autophagy (Yu *et al.*, 2020 ▸). In *fsc*1Δ mutant cells, mature autophagosomes are able to establish contact with the vacuole membrane; however, membrane fusion does not occur. This phenotype suggests that while the HOPS complex and SNARE proteins are sufficient to facilitate organelle docking, Fsc1 is required for complete membrane merger (Sun *et al.*, 2013 ▸). A *BLAST* search identified an Fsc1 homologous protein in *S. cerevisiae*, Ylr001c (Priyam *et al.*, 2019 ▸); however, Ylr001c lacks a defined role in autophagy (Sun *et al.*, 2013 ▸), suggesting an evolutionary divergence between the two yeasts.

Proteins in the fasciclin superfamily typically contain multiple FAS1 domains and adopt elongated, scaffold-like architectures, which accommodate multiple binding sites (Seifert, 2018 ▸). Across diverse organisms, FAS1-containing proteins use their elongated surfaces to mediate biological functions through interactions with partner molecules. In humans, periostin contains four tandem FAS1 domains that serve as a recruitment scaffold for collagen I, fibronectin and tenascin-C, thereby linking the extracellular matrix to cell-surface signaling during organogenesis (Rusbjerg-Weberskov *et al.*, 2024 ▸; Kii & Ito, 2017 ▸). Similarly, FAS1 domains in human TGFBI bind integrins, thereby mediating cell adhesion that is essential for ocular tissue maintenance and vision (Son *et al.*, 2013 ▸). In other systems such as the microalga astaxanthin-binding protein AstaP, the FAS1 domain functions as a clamp-like structure that captures carotenoids (Kornilov *et al.*, 2023 ▸). Notably, Fsc1 contains five tandem FAS1 domains, the largest number among structurally characterized fasciclin proteins, and localizes to the vacuole, suggesting that its FAS1 repeats may facilitate autophagy through scaffold-mediated inter­actions with potential binding partners, as observed for other members of the fasciclin family.

Despite its essential role in autophagosome–vacuole fusion, the structural basis of Fsc1 function has remained unknown. Here, we report the first crystal structures of the soluble lumenal domain of *S. pombe* Fsc1 and provide a comprehensive analysis of its domain organization, oligomeric state and conserved structural features. These results establish a structural framework for understanding how the modular FAS1 architecture of Fsc1 may contribute to late-stage membrane fusion during autophagy.

## 2. Materials and methods

### 2.1. Cloning and expression

The plasmid encoding *S. pombe* Fsc1 was obtained from Dr Li-Lin Du of the National Institute of Biological Sciences, Beijing, China. To generate an expression construct for the soluble region of Fsc1, the corresponding coding sequence was subcloned into the pSMT3 expression vector using XhoI and BamHI restriction sites, with forward (5′-AGAGAACAGATTGGTGGAATGAACCTTCAATTTCGG-3′) and reverse (5′-GTGGTGGTGGTGGTGCTCGAGCTAAGTTATTCTCCAACGATTTTG-3′) primers. The resulting construct included an N-terminal His-tag and SUMO fusion to facilitate purification and efficient removal of the affinity tag, enabling the recovery of native Fsc1. The plasmid was transformed into *Escherichia coli* Top10 cells for amplification.

For protein expression, the pSMT3-Fsc1 plasmid was transformed into *E. coli* Rosetta cells. Transformed colonies were selected on LB–agar plates supplemented with 34 µg ml^−1^ chloramphenicol and 50 µg ml^−1^ kanamycin and incubated overnight at 37°C. Single colonies were used to inoculate a 25 ml starter culture, which was grown at 37°C for 3 h and subsequently expanded into 1 l LB medium with the same antibiotics. Protein expression was induced at an OD_600_ of ∼0.6 by the addition of 100 µ*M* IPTG followed by incubation at 16°C for 16 h. For selenomethionine (SeMet)-substituted Fsc1, cells were grown in M9 minimal medium using the same induction protocol.

### 2.2. Protein purification

Harvested cells were resuspended in lysis buffer (20 m*M* Tris pH 8.0, 500 m*M* NaCl, 10 m*M* imidazole) supplemented with PMSF protease inhibitor and lysed by five cycles of sonication using a Branson Sonifier 450. The lysate was cleared by centrifugation at 15 000 rev min^−1^ for 30 min at 4°C. The supernatant was incubated with 4 ml Ni–NTA resin pre-equilibrated in lysis buffer at 4°C for 1 h. After washing with lysis buffer followed by wash buffer containing 50 m*M* imidazole, bound Fsc1 was eluted with buffer containing 300 m*M* imidazole. The His-SUMO tag was cleaved overnight at 4°C using Ulp1 protease, and successful cleavage and protein purity were confirmed by SDS–PAGE.

Further purification was performed by sequential chromatography steps using an ÄKTApure chromatography system (Cytiva). The protein was first subjected to ion-exchange chromatography on a Resource Q column (Cytiva) using a linear NaCl gradient generated by mixing buffer *A* (50 m*M* NaCl, 20 m*M* Tris pH 8.0) and buffer *B* (1 *M* NaCl, 20 m*M* Tris pH 8.0). Fractions containing Fsc1 were pooled and further purified by size-exclusion chromatography on a Superdex 200 column (Cytiva) in SEC buffer (20 m*M* Tris pH 8.0, 150 m*M* NaCl). Purified fractions were concentrated and used for crystallization.

### 2.3. Blue native PAGE

The oligomeric state of the Fsc1 construct was assessed by blue native PAGE (Wittig *et al.*, 2006 ▸; Na Ayutthaya *et al.*, 2020 ▸) using Novex 4–16% Bis-Tris gels (Invitrogen). Bovine serum albumin (BSA) was included as a molecular-weight marker. Gels were run at 4°C and stained with Coomassie Brilliant Blue, and the apparent molecular weight of Fsc1 was estimated by comparison with the migration of known markers to infer its dimeric state.

### 2.4. Crystallization and data collection

Initial crystallization screening using sitting-drop vapor diffusion identified two crystal forms, designated *A* (space group *C*2) and *B* (space group *P*4_3_2_1_2). Crystallization conditions were subsequently optimized using hanging-drop vapor diffusion at a protein concentration of 3.5 mg ml^−1^. Form *A* crystals were obtained in 11%(*w*/*v*) PEG 3350, 0.4 *M* MgCl_2_, 0.1 *M* Tris pH 8.5. Form *B* crystals formed in 8%(*w*/*v*) PEG 3350, 0.2 *M* MgCl_2_, 0.1 *M* Tris pH 8.5. Crystals were cryoprotected with 30%(*v*/*v*) ethylene glycol, flash-cooled in liquid nitrogen and diffraction data were collected on the 21-ID-D beamline at the Advanced Photon Source, Argonne National Laboratory. Data were indexed, integrated and scaled using *HKL*-2000 (Otwinowski & Minor, 1997 ▸).

### 2.5. Structure determination and refinement

Initial phases were determined by single-wavelength anomalous dispersion (SAD) using data collected from SeMet-labeled form *B* crystals. The resulting model was subsequently used as a search model for molecular replacement to solve the structure of form *A* crystals using *Phenix* (Liebschner *et al.*, 2019 ▸). Model rebuilding and manual inspection, including the placement of water molecules, were performed in *Coot* (Emsley *et al.*, 2010 ▸). Iterative structural refinement was carried out using *phenix.refine* in *Phenix* and *REFMAC*5 from the *CCP*4 suite (Murshudov *et al.*, 2011 ▸; Agirre *et al.*, 2023 ▸). The final models were validated with *MolProbity* in *Phenix*. The refinement and validation statistics are summarized in Table 1 ▸. The coordinates and structural factors have been deposited in the Protein Data Bank (PDB) under the PDB codes listed in Table 1 ▸.

### 2.6. Multiple sequence alignment and structure analysis

*DELTA-BLAST* searches were performed using *Protein-BLAST* (Altschul *et al.*, 1990 ▸) to identify (i) additional fasciclin-containing proteins in *S. pombe*, (ii) Fsc1-related autophagy proteins in *S. pombe* and (iii) homologous FAS-1 domain-containing proteins. Multiple sequence alignment was performed using *ClustalW* (Sievers *et al.*, 2011 ▸), and protein identity and similarity calculations and placement were performed using the protein *SIAS* server (Universidad Complutense Madrid; https://imed.med.ucm.es/Tools/sias.html). Structural alignments were performed using *ESPript* v3.0 (Robert & Gouet, 2014 ▸). Protein–protein interface analyses and association-state predictions were performed using *PDBePISA* (Krissinel & Henrick, 2007 ▸). Structural conservation weighted analysis was performed with *ConSurf* (Ashkenazy *et al.*, 2016 ▸; Glaser *et al.*, 2003 ▸), and electrostatic surface potentials were calculated using the *PDB*2*PQR* and *APBS* electrostatics web servers at pH 5.5 to mirror the vacuolar environment (Gachet *et al.*, 2005 ▸), with default parameters and a potential range of ±5 *kT*/*e*, where *k* is the Boltzmann constant, *T* is the temperature in kelvin and *e* is the elementary charge (Jurrus *et al.*, 2018 ▸). Structural visualization and figure generation were performed in *PyMOL* (DeLano, 2010 ▸). The full-length Fsc1 protein structure predicted by *AlphaFold*2 was obtained from the AlphaFold Database (AF ID O94439). The short transmembrane and cytosolic regions of Fsc1 were modeled using the *AlphaFold*2-predicted structure (Jumper *et al.*, 2021 ▸).

## 3. Results

### 3.1. Overall structure of Fsc1

**Figure 1:**
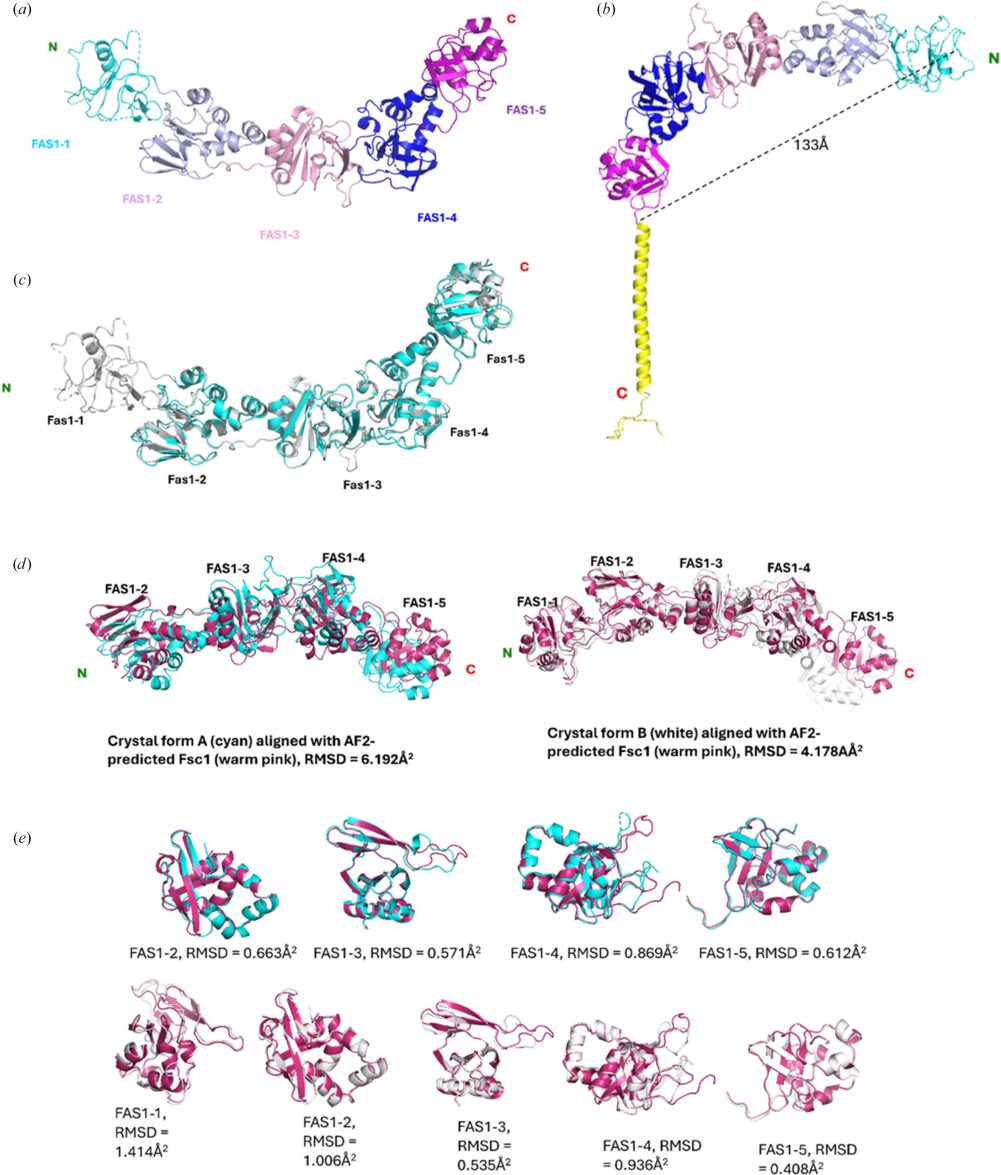
Overall architecture and structural comparison of Fsc1. (*a*) Ribbon diagram of the Fsc1 monomer showing the five tandem FAS1 domains (FAS1-1 to FAS1-5), colored sequentially from the N-terminus to the C-terminus. (*b*) Domain organization of Fsc1, highlighting the continuous fasciclin repeat architecture without intervening motifs, and showing the short single-pass transmembrane and vacuolar domain in yellow (predicted by *AlphaFold*); the coloring scheme is consistent with that in (*a*). (*c*) Superposition of crystal forms *A* (cyan) and *B* (gray), showing structural consistency with minor variations. R.m.s.d. = 0.649 Å. (*d*) Global comparison of the *AlphaFold*-predicted Fsc1 structure (in warm pink) to experimental structures of forms *A* (cyan, r.m.s.d. = 6.192 Å) and *B* (gray, r.m.s.d. = 4.178 Å). (*e*) Pairwise structural comparison of individual FAS1 domains between *AlphaFold*-predicted Fsc1 and both crystal forms; the coloring scheme is consistent with (*d*).

The full-length Fsc1 protein from *S. pombe* is an 82.6 kDa single-pass transmembrane vacuolar protein comprising 728 amino acids, arranged into a large lumenal region (Lys1–Arg647), a short single-pass transmembrane domain (Ile648–Tyr670) and a cytosolic region (Phe671 to the C-terminus) (Sun *et al.*, 2013 ▸; Hallgren *et al.*, 2022 ▸). To characterize the luminal domain, we cloned and expressed an Fsc1 construct encompassing residues 1–649 (∼72 kDa) in *E. coli* and determined its structure in two distinct crystal forms (form *A*, space group *C*2; form *B*, space group *P*4_3_2_1_2) using a combination of single anomalous diffraction and molecular replacement (see Table 1 ▸). Despite differences in crystal symmetry (*C*2 versus *P*4_3_2_1_2), the structures from both crystal forms exhibit a consistent overall architecture, with a root-mean-square deviation (r.m.s.d.) of 0.649 Å between the two models (Figs. 1 ▸*a*–1 ▸*c*). This strong agreement between two independent crystal forms underscores the robustness and reliability of the structural features described below.

The Fsc1 structure reveals five consecutive fasciclin-1 (FAS1) domains: FAS1-1 (Ile11–Asp131), FAS1-2 (Leu132–Lys246), FAS1-3 (Thr272–Leu388), FAS1-4 (Pro398–Lys533) and FAS1-5 (Gln542–Lys641) (Fig. 1 ▸*a*). These domains are arranged in a continuous, linear fashion without intervening motifs, forming a slightly curved, elongated, modular architecture approximately 133 Å in length (Fig. 1 ▸*b*). This extended configuration generates a considerably large surface area that is well suited for interactions with potential binding partners. In crystal form *A*, electron density was insufficient to model several regions of the FAS1-1 domain, particularly in the N-terminal region. Crystal form *B* exhibited improved density overall; however, portions of the FAS1-1 domain remained disordered and were therefore excluded from the final model. Consistent with these observations, the *AlphaFold*-predicted model also indicates pronounced flexibility in this region, as reflected by the predicted local distance difference test (pLDDT) scores (Supplementary Fig. S1). pLDDT values represent *AlphaFold*2’s confidence in local structural predictions, with scores below 70 commonly associated with intrinsically disordered or highly flexible regions (Jumper *et al.*, 2021 ▸). In the predicted Fsc1 model, pLDDT scores fall below 70 within the first 100 residues, corresponding to the FAS1-1 domain, as well as the region beyond residue 650 downstream of FAS1-5 (Supplementary Fig. S1). The observed conformational flexibility of the FAS1-1 domain suggests a potential role in dynamic, transient or regulated interactions.

Comparison of the *AlphaFold*-predicted structure of Fsc1 with the experimentally determined structures presented here reveals a clear distinction between local and global organization. Global alignment of the predicted model with crystal forms *A* and *B* yields r.m.s.d. values of 6.19 and 4.18 Å^2^ (Fig. 1 ▸*d*), respectively, indicating overall similarity but notable differences in interdomain arrangement. In contrast, pairwise comparisons of individual FAS1 domains between the predicted and experimental structures show strong local agreement, with minimal backbone deviations and r.m.s.d. values ranging from 0.41 to 1.41 Å^2^ (Fig. 1 ▸*e*). Consistent with these observations, *AlphaFold*2 predicted aligned error (PAE) plots further indicate high confidence in the folding of individual domains but low confidence in their relative orientations, supporting the presence of substantial interdomain flexibility rather than a single rigid global conformation (Jumper *et al.*, 2021 ▸; Supplementary Fig. S1). Thus, while *AlphaFold* accurately predicts the structures of individual FAS1 domains, it fails to capture the experimentally observed global architecture of Fsc1, instead favoring a more linear interdomain arrangement.

### 3.2. Domain architecture

**Figure 2:**
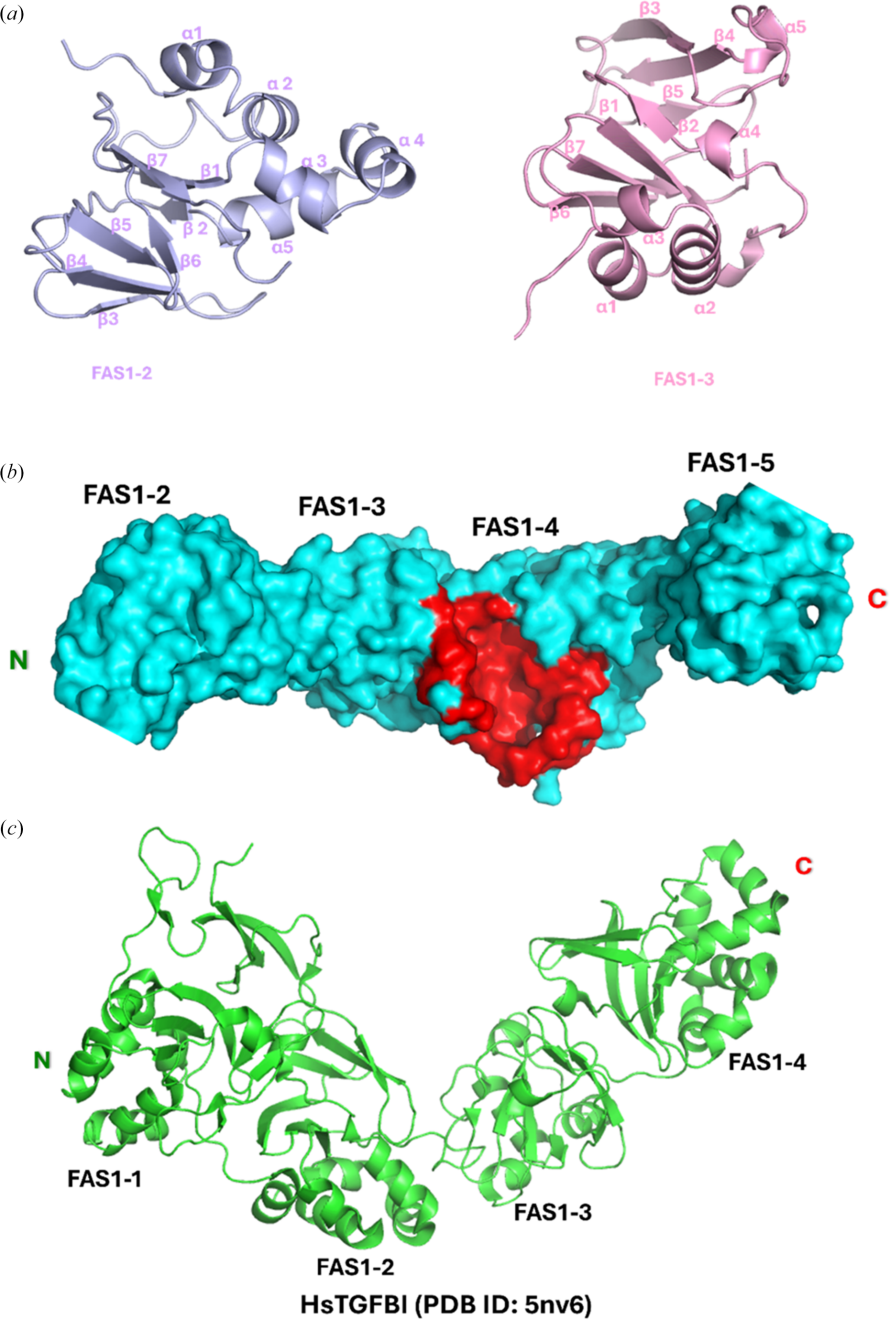
Domain architecture and structural features of Fsc1 FAS1 domains. (*a*) Representative β-sandwich fold of Fsc1. A detailed view of FAS1-2 and FAS1-3, with secondary-structure elements annotated to highlight the β-strands and α-helices characteristic of the fasciclin fold. (*b*) Surface representation of Fsc1, highlighting a deep central groove present in FAS1-4 (residues Thr518–Asp532) shown in red. (*c*) FAS1 domains of human TGFBI (PDB entry 5nv6), featuring expanded β-sheets and α-helices (six to seven elements) compared with the five or six β-sheet/α-helix elements typically observed in most fasciclin proteins.

Each Fsc1 FAS1 domain adopts a jelly-roll β-sandwich fold, a core structural motif commonly observed in adhesion proteins (Chothia & Jones, 1997 ▸), flanked by an α-helical region composed of multiple right-handed α-helices (Figs. 1 ▸*a*–1 ▸*c*). Across the fasciclin family, FAS1 domains typically comprise a β-sandwich formed by approximately six β-strands, arranged as two three-stranded sheets angled relative to one another and bordered by an α-helical bundle of roughly six helices arranged in a V-shaped hairpin configuration (Seifert, 2018 ▸; Liu *et al.*, 2018 ▸; Twarda-Clapa *et al.*, 2018 ▸; García-Castellanos *et al.*, 2017 ▸; Underhaug *et al.*, 2013 ▸). This mixed α-helix/β-strand architecture is exemplified by the FAS1-2 and FAS1-3 domains (Fig. 2 ▸*a*), each of which contains five prominent α-helices flanking two antiparallel β-sheets. The FAS1-4 domain introduces a pronounced ∼125° bend relative to FAS1-3, forming an ‘elbow’ in the molecular backbone (Fig. 1 ▸*a*). This bend, together with a large surface-exposed groove spanning residues Thr518–Asp532 (Fig. 2 ▸*b*), may provide a potential interface for protein–protein interactions or complex assembly. The C-terminal FAS1-5 domain exhibits a similar organization, with short α-helices concentrated near the N-terminus of the domain and a β-sandwich core composed of five antiparallel β-strands.

The overall architecture of Fsc1 is characteristic of the fasciclin superfamily and is consistent with previously reported structures of other FAS1-containing proteins from diverse organisms (Seifert, 2018 ▸; Kornilov *et al.*, 2023 ▸). Although modest variation exists in the number of β-strands and α-helices among fasciclin proteins, the core fold is highly conserved. For example, the FAS1 domains of human TGFBI (PDB entry 5nv6) adopt a β-sandwich core composed of six to seven β-strands flanked by an α-helical bundle of five α-helices (García-Castellanos *et al.*, 2017 ▸; Nielsen *et al.*, 2021 ▸; Fig. 2 ▸*c*).

Although these TFGBI folds contain an additional β-strand relative to some fasciclin family members, such modest variations in α-helix/β-strand composition have not been shown to be physiologically significant across the fasciclin superfamily.

### 3.3. Dimerization of Fsc1

**Figure 3:**
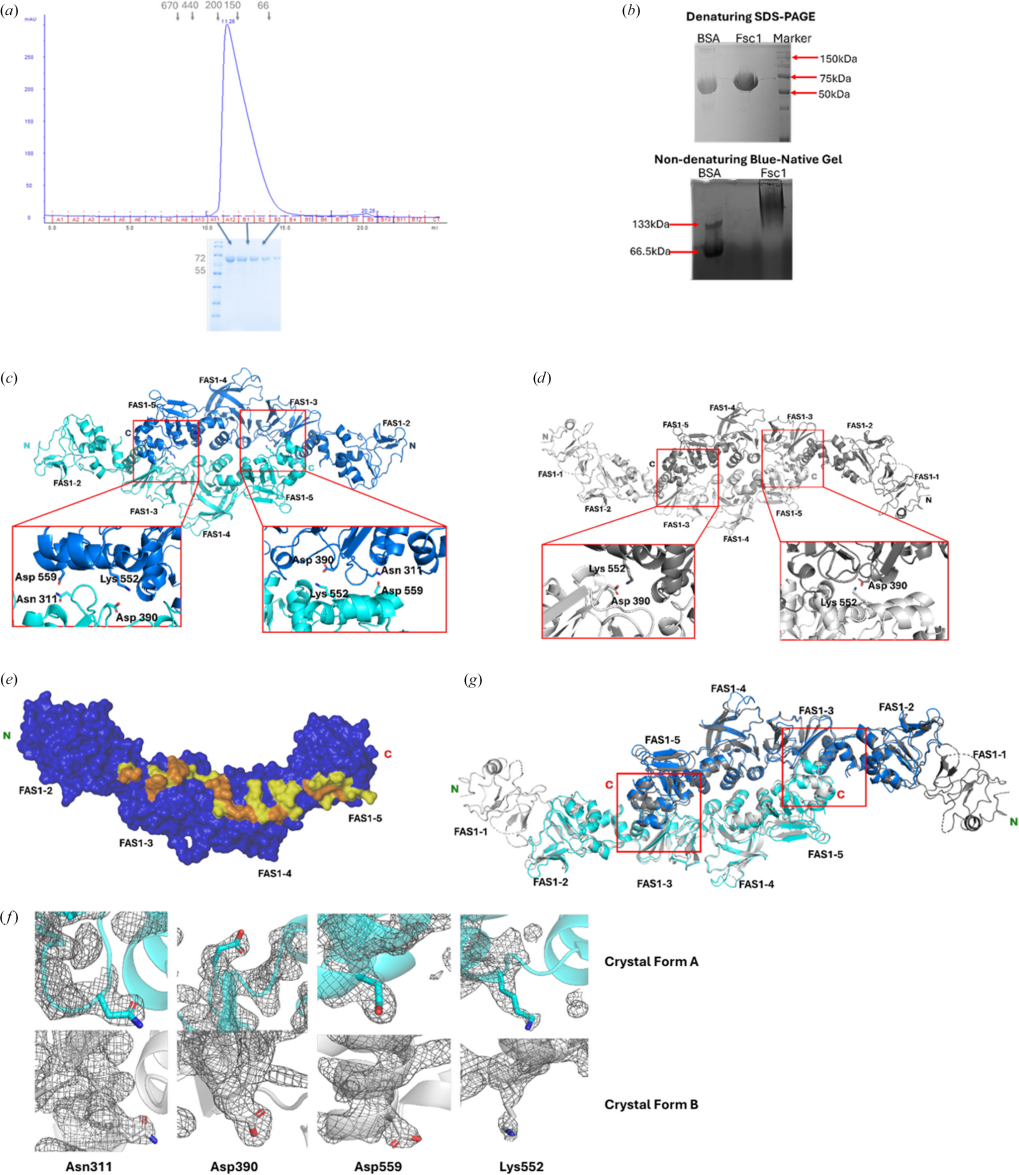
Oligomeric state and interfacial analysis of Fsc1. (*a*) Size-exclusion chromatography (SEC) profile of purified Fsc1 showing a predominant dimeric species (∼145 kDa). The corresponding molecular weights (in kDa) are indicated on both the chromatogram and the SDS–PAGE gel. (*b*) Denaturing SDS–PAGE and blue native PAGE of the Fsc1 construct. Bovine serum albumin (BSA) was included as a marker for the native gel run. (*c*) Dimer interface in crystal form *A*, with key residues involved in hydrogen bonding and salt-bridge formation highlighted. (*d*) Dimer interface in crystal form *B*, showing similar interactions to (*c*), with the key residues highlighted. (*e*) Surface properties of the Fsc1 dimerization interface. One protomer (dark blue) is shown with interface residues colored by chemical property: hydrophilic (yellow) and hydrophobic (orange). (*f*) Representative electron density at the dimer interface. The 2*F*_o_ − *F*_c_ map (contoured at 1.5σ) shows well defined density for polar side chains involved in the interactions. (*g*) Superimposition of the dimer interfaces from both crystal forms, as depicted in (*c*) and (*d*), reveals a consistent topology that supports biological relevance.

Size-exclusion chromatography (SEC) indicates that Fsc1 exists predominantly as a dimer in solution, eluting at a volume corresponding to an apparent molecular mass of approximately 145 kDa, consistent with a dimer composed of ∼72 kDa monomers (Fig. 3 ▸*a*). To further assess the oligomeric states of the Fsc1 construct, we performed blue native PAGE (BN-PAGE). Bovine serum albumin (BSA), which exists as a mixture of monomeric and dimeric species with monomers predominating, was used as a molecular-weight marker (Pandhare *et al.*, 2019 ▸). Under native conditions, Fsc1 migrated at an apparent molecular weight greater than 133 kDa on BN-PAGE (Fig. 3 ▸*b*), consistent with a dimeric species, compared with the ∼72 kDa monomer observed under SDS denaturing conditions. This solution-based dimerization is further supported by structural data and crystal-packing analyses, which reveal a common dimerization interface present in both crystal forms, suggesting a biologically relevant interaction (Figs. 3 ▸*c* and 3 ▸*d*). *PISA* interface analysis further shows that in each crystal form only a single interface exhibits a buried surface area (BSA) exceeding 970 Å^2^, an empirical threshold associated with stable biological dimers (Bahadur *et al.*, 2003 ▸; Krissinel & Henrick, 2007 ▸). These interfaces were therefore selected for further structural analysis, as described below.

In both crystal forms, the dimerization interface is formed by symmetric, antiparallel interactions between domains FAS1-3, FAS1-4 and FAS1-5 of each monomer (Figs. 3 ▸*c* and 3 ▸*d*). Each Fsc1 protomer contributes complementary interdomain contacts between interacting protomers *X* and *Y*: FAS1-3_*X*_:FAS1-5_*Y*_, FAS1-4_*X*_:FAs1-4_*Y*_ and FAS1-5_*X*_:FAS1-3_*Y*_. In crystal form *A*, this interface buries approximately 1110 Å^2^ of surface area, while crystal form *B* displays a similar interface with a buried surface area of ∼1142 Å^2^. The minimal difference in buried surface area between the two forms is most likely attributable to crystal-packing effects rather than biologically meaningful differences between the two crystal forms. The Fsc1 dimer interface is mediated by a network of electrostatic interactions and hydrophobic contacts (Fig. 3 ▸*e*). Interface residues were defined as those containing at least one heavy atom within 5 Å of the opposing protomer and are further elucidated below. The electrostatic interactions maintaining this dimer interface include reciprocal hydrogen bonds formed between the side chains of residues Asn311 and Asp559, contributed by the FAS1-3 and FAS1-5 domains of opposing monomers (Fig. 3 ▸*c*). Although the overall orientation of these interactions is consistent across both forms, minor interatomic variations are observed. In addition, two interchain salt bridges between the ɛ-amino group of Lys552 and the carboxylate side chain of Asp390 on opposing Fsc1 monomers further stabilize this dimer interface (Fig. 3 ▸*c*). These polar interactions are supported by well defined electron density (as shown in Fig. 3 ▸*f*). Structural superposition of the dimer assemblies from both crystal forms reveals a highly conserved interface geometry (Fig. 3 ▸*g*), supporting the biological relevance of the observed Fsc1 dimer assembly.

Although *PISA* analysis indicates that the Fsc1 dimer interface buries a relatively modest surface area, the consistent observation across SEC and BN-PAGE assays support the stability of this assembly in solution. Electrostatic surface mapping reveals that Fsc1 is predominantly polar (Supplementary Fig. S2), suggesting that this interface may be non-obligate and instead regulated in a context-dependent manner (Yan *et al.*, 2008 ▸). Together, these observations are consistent with a low-affinity yet biologically relevant transient dimer, a characteristic commonly observed in protein superfamilies whose functions involve a mechanism of action through some form of adhesion (Wu *et al.*, 2010 ▸). Notable examples include cadherins (Honig & Shapiro, 2020 ▸) and integrins (Jun *et al.*, 2001 ▸).

### 3.4. Conserved motifs and comparative analysis

**Figure 4:**
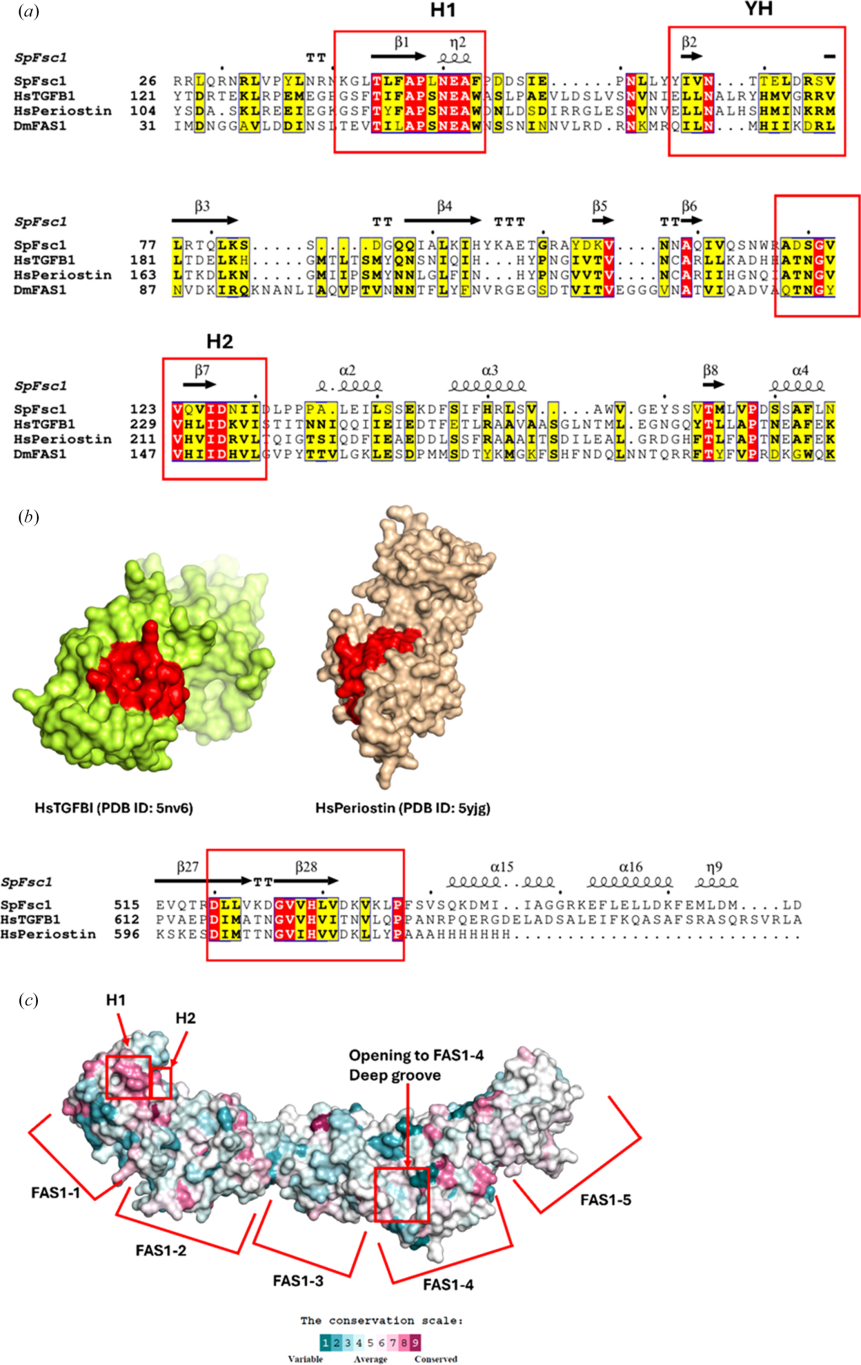
Sequence conservation and comparative surface topography of FAS1 domains. (*a*) Multiple sequence alignment of Fsc1 and human fasciclin proteins TGFBI and periostin, highlighting the conserved H1, YH and H2 motifs. (*b*) Comparative surface analysis of human homologs. Surface representations of TGFBI (lemon; PDB entry 5nv6) and periostin (wheat; PDB entry 5yjg) are shown, with regions corresponding to the Fsc1 FAS1-4 groove highlighted in red, demonstrating conserved surface topology across species as corroborated by the sequence alignment. (*c*) *ConSurf* analysis and conservation mapping of Fsc1, with the molecular surface colored according to evolutionary conservation score. Key features, including the H1 and H2 motifs and the FAS1-4 groove, are labeled to highlight their localization within highly conserved regions.

Multiple sequence alignment (MSA) and *DELTA-BLAST* analyses identified Fsc1 as the sole fasciclin-containing protein in *S. pombe*. However, homologous FAS1-containing proteins were detected in other organisms, including human TGFBI and periostin. The MSA revealed the conservation of hallmark fasciclin motifs in Fsc1, including H1 (Leu43–Phe53), the YH motif (Tyr63–Thr79) and H2 (Ala118–Ile130), which have previously been associated with structural stability and ligand recognition (Moody & Williamson, 2013 ▸; Kim *et al.*, 2002 ▸; Fig. 4 ▸*a*).

Structural alignment using *DALI* confirmed significant similarity between the FAS1 domains of Fsc1 and those of TGFBI and periostin, with the strongest correspondence observed in FAS1-4 (Holm, 2022 ▸). Notably, the pronounced surface groove in the Fsc1 FAS1-4 domain overlaps with conserved depressions in the corresponding domains of TGFBI and periostin (Fig. 4 ▸*b*; García-Castellanos *et al.*, 2017 ▸; Liu *et al.*, 2018 ▸). Consistent with this observed structural similarity, *ConSurf* analysis revealed strong conservation within the groove-forming region (Asp520–Pro537; Figs. 4 ▸*b* and 4 ▸*c*; Supplementary Fig. S3). This region does not participate in Fsc1 dimerization, and the precise role of this conserved surface in Fsc1-mediated fusion remains unclear; however, it may represent a conserved interface for inter­actions with partners besides an opposing Fsc1 monomer, as suggested for other FAS1-containing proteins (Liu *et al.*, 2018 ▸).

## 4. Discussion

In this work, we determined the first crystal structures of Fsc1, revealing an architecture composed of five tandem FAS1 domains, the largest number of consecutive FAS1 domains structurally characterized to date. Although the fasciclin superfamily lacks a universal ligand or family-wide binding partner, prior biochemical studies indicate that FAS1-containing proteins commonly function as scaffolds that organize interacting partners to mediate their biological roles (Kornilov *et al.*, 2023 ▸). By analogy, the elongated, modular arrangement of Fsc1 FAS1 domains suggests that Fsc1 may serve as a scaffold that coordinates the multiple interactions required for complete autophagosome–vacuole membrane fusion.

Membrane fusion is widely described by the stalk–hemifusion model, which proposes a series of energetically coupled steps beginning with membrane tethering, followed by hemifusion of the proximal lipid leaflets, fusion-pore formation and pore expansion to yield a single continuous membrane (Chernomordik & Kozlov, 2005 ▸, 2008 ▸; Jahn *et al.*, 2003 ▸). In the context of autophagosome–vacuole fusion, tethering is mediated by the HOPS complex, which brings the two membranes into close apposition (Shvarev *et al.*, 2022 ▸; Chernomordik & Kozlov, 2005 ▸, 2008 ▸; Jahn *et al.*, 2003 ▸). This is followed by hemifusion, in which the proximal leaflets of the lipid bilayers merge, generating mechanical stress at the fusion site (Chernomordik & Kozlov, 2005 ▸). Subsequent formation of a fusion pore enables lipid and protein exchange between the apposed membranes, and progressive pore expansion ultimately remodels the membrane barrier to yield a single fused organelle (Scherer *et al.*, 2026 ▸; Reese & Mayer, 2005 ▸). Given the conserved nature of biological membrane fusion and the strong experimental support for the stalk–hemifusion model, these stepwise processes are likely to underlie autophagosome–vacuole membrane fusion in *S. pombe*.

Although the precise molecular events that drive membrane fusion during autophagy remain incompletely understood, genetic studies provide important functional constraints. In *fsc*1Δ cells, autophagosomes dock at the vacuolar membrane but fail to fuse (Sun *et al.*, 2013 ▸), indicating that Fsc1 is dispensable for tethering but essential for downstream membrane-fusion events. Within the framework of the stalk–hemifusion model, Fsc1 is therefore likely to act at a narrow post-docking stage of the fusion pathway. Fsc1 may contribute to membrane fusion by promoting the membrane curvature or tensile stress necessary for bilayer merger following HOPS-mediated docking, or it may facilitate the energetically favorable perturbations required for initial fusion-pore formation and pore expansion (Chernomordik & Kozlov, 2005 ▸; Martens & McMahon, 2008 ▸). Alternatively, Fsc1 could promote lipid and protein exchange across the apposed autophagosome–vacuole membranes, thereby enabling the membrane remodeling necessary for complete fusion (Chernomordik & Kozlov, 2005 ▸). These possibilities are not mutually exclusive and may reflect distinct roles of Fsc1 at successive stages of the fusion process.

Our biochemical and structural analyses further demonstrate that Fsc1 forms a homodimer in solution. However, the absence of a conserved oligomerization state across the fasciclin superfamily (Liu *et al.*, 2018 ▸; Twarda-Clapa *et al.*, 2018 ▸; García-Castellanos *et al.*, 2017 ▸), together with the modest buried surface area, predominantly polar character and limited conservation of residues at the dimer interface, suggests that Fsc1 self-association is likely to be of low affinity *in vivo.* Such dimerization may therefore be transient or condition-dependent, potentially stabilized only during specific stages of autophagy or upon interaction with binding partners. This hypothesis can be tested by targeted mutagenesis designed to disrupt the dimer interface, followed by functional analysis of these mutants in *S. pombe*. Observation of fusion defects phenocopying *fsc1Δ* cells would provide strong evidence for a context-dependent functional role of Fsc1 dimerization during autophagy.

In addition to its oligomeric properties, Fsc1 contains several conserved structural features, including the canonical fasciclin H1, H2 and YH motifs, as well as a deep surface groove within the FAS1-4 domain that contains conserved residues. Notably, the H1 and H2 motifs are located within the FAS1-1 domain (Fig. 4 ▸*c*), for which electron density is poorly defined and *AlphaFold* confidence scores are low, suggesting pronounced conformational flexibility. While the functional significance of this flexibility remains unclear, the coexistence of conserved sequence motifs and structural plasticity in FAS1-1 is consistent with a potential role in regulated or transient interactions, a hallmark of intrinsically flexible protein regions involved in signaling and membrane-remodeling processes (Bondos *et al.*, 2022 ▸). In contrast, the conserved FAS1-4 surface groove does not participate in homodimer formation and may represent a binding site for other components of the autophagy fusion machinery.

In summary, our study defines the structural organization of Fsc1 and provides a framework for understanding its function in autophagosome–vacuole membrane fusion. The tandem FAS1-domain architecture, conserved interaction motifs and context-dependent dimerization properties together support a model in which Fsc1 acts as a modular scaffold that facilitates late-stage membrane-fusion events. Future studies integrating targeted mutagenesis, biochemical interaction assays and cellular analyses will be essential to identify Fsc1 binding partners and to elucidate how Fsc1 coordinates the molecular machinery that drives autophagosome–vacuole fusion during autophagy.

## Supplementary Material

PDB reference: fission yeast Fsc1 protein, space group *C*2, 9nu9

PDB reference: space group *P*4_3_2_1_2, 9o0b

Supplementary Figures. DOI: 10.1107/S205979832600197X/jb5070sup1.pdf

## Figures and Tables

**Table 1:** Data-collection and refinement statistics Values in parentheses are for the outer shell.

	Fsc1 crystal form *A*	Fsc1 crystal form *B*
Data collection		
Resolution range	47.84–2.45 (2.54–2.45)	41.01–2.50 (2.59–2.50)
Space group	*C*2	*P*4_3_2_1_2
*a*, *b*, *c* (Å)	347.82, 40.91, 59.10	58.00, 58.00, 488.27
α, β, γ (°)	90.00, 92.57, 90.00	90.00, 90.00, 90.00
Unique reflections	29304 (1910)	29781 (2829)
Completeness (%)	93.85 (62.13)	97.83 (96.09)
Wilson *B* factor (Å^2^)	33.55	53.27
Refinement		
Reflections used in refinement	29292 (1910)	29781 (2829)
Reflections used for *R*_free_	1458 (104)	1968 (189)
*R*_work_	0.2053 (0.2608)	0.2390 (0.3184)
*R*_free_	0.2505 (0.2990)	0.2971 (0.3404)
No. of non-H atoms		
Total	4254	4965
Macromolecules	4018	4853
Ligands	0	0
Solvent	236	112
Protein residues	507	609
R.m.s.d., bond lengths (Å)	0.01	0.008
R.m.s.d., angles (°)	1.33	1.58
Ramachandran favored (%)	92.28	95.1
Ramachandran allowed (%)	7.72	4.9
Ramachandran outliers (%)	0	0
Rotamer outliers (%)	5.11	7.17
Clashscore	9.51	12.79
Average *B* factor (Å^2^)		
Overall	58.84	66.06
Macromolecules	59.6	66.36
Solvent	46.02	52.88
PDB code	9nu9	9o0b
