# Supplementary material for: Crystal structures of Fsc1, a novel autophagy factor that mediates autophagosome–vacuole fusion in fission yeast
Source: Acta Crystallogr D Struct Biol. 2026 Mar 25;82(Pt 4):358–69. doi: 10.1107/S205979832600197X (PMC13044898; doi:10.1107/S205979832600197X)
Supplement: Supplementary file 1 [file d-82-00358-sup1.pdf]

STRUCTURAL BIOLOGY

Volume 82 (2026)

Supporting information for article:

Crystal structures of Fsc1, a novel autophagy factor that mediates autophagosome–vacuole fusion in fission yeast

Xiangshu Jin, Chidiogo Azuka and Liu Jian

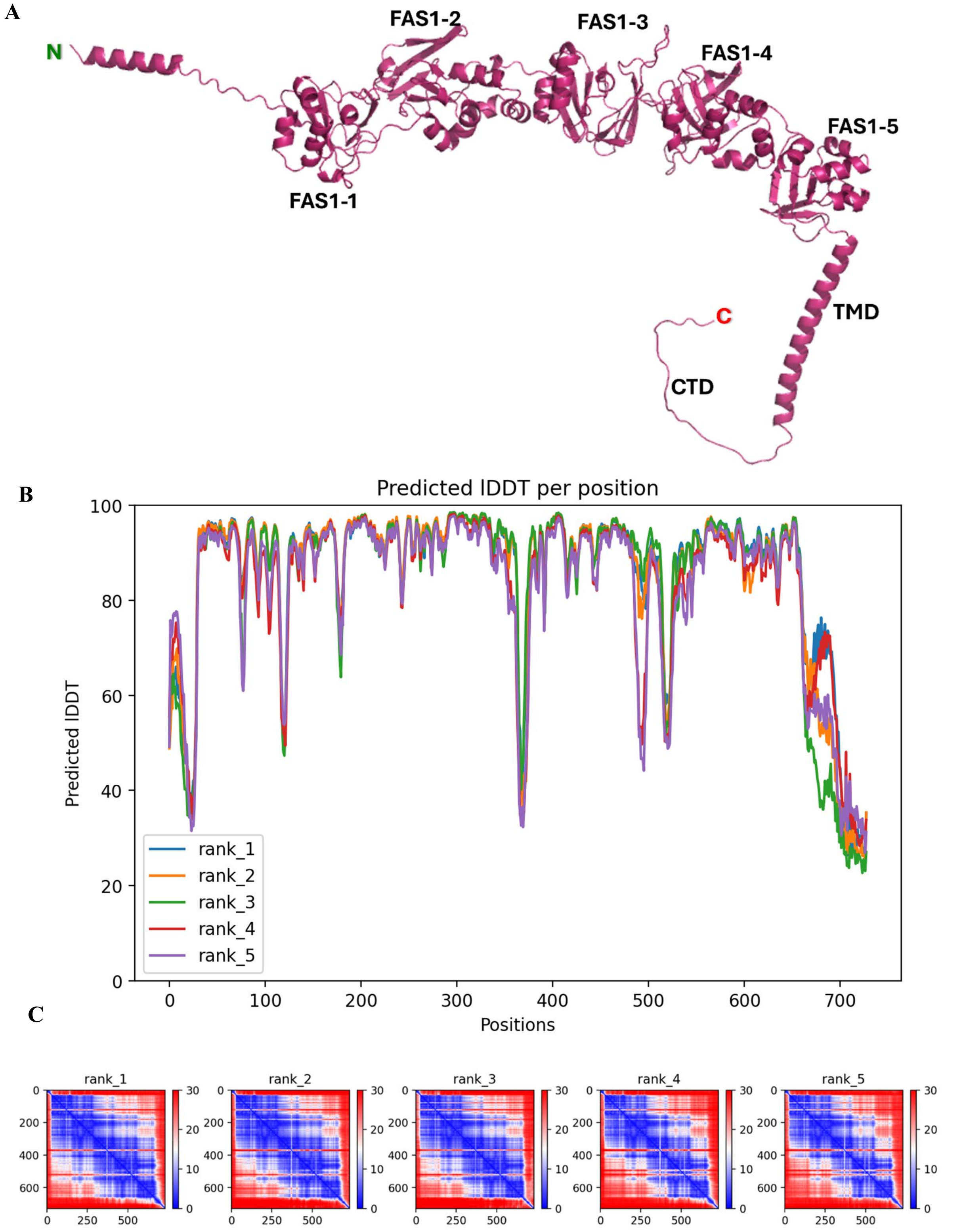
A
N
FAS1-2
FAS1-3
FAS1-4
FAS1-5
FAS1-1
TMD
C
CTD
B
Predicted lDDT per position
Predicted lDDT
Positions
rank_1
rank_2
rank_3
rank_4
rank_5
C
rank_1
rank_2
rank_3
rank_4
rank_5

Figure S1 AlphaFold2 structural model and prediction confidence and error metrics for full-length Fsc1. **(**A) Predicted structure of three-dimensional structure of full-length Fsc1 generated by AlphaFold2. (B) Per-residue confidence scores (pLDDT; predicted Local Distance Difference Test) scores. Regions with high confidence regions (pLDDT > 90) are predicted with high accuracy, ehereas regions with lower scores (<70) likely correspond to flexible or intrinsically disordered segments. (C) Predicted Aligned Error (PAE) plot, showing the estimated positional error between all residue pairs, which reflects the relative confidence in inter-domain and intra-domain arrangements.

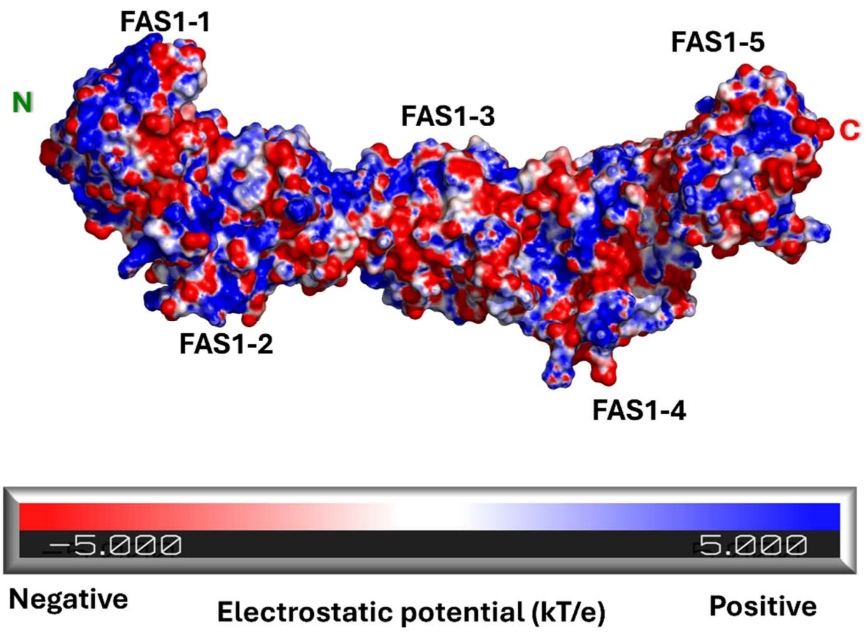

Figure S2 Surface electrostatic potential of Fsc1 FAS1 domains.

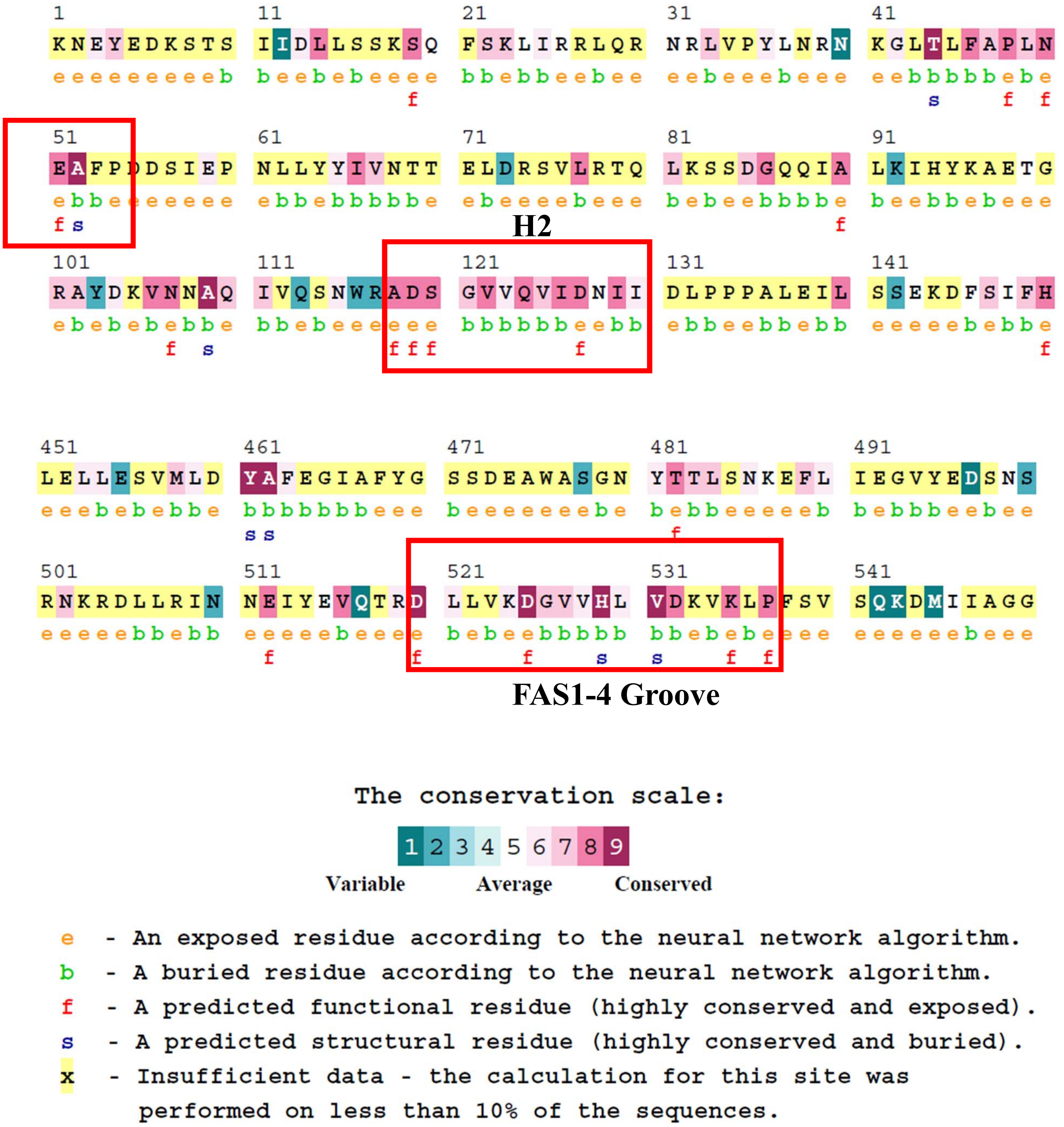

Figure S3 Evolutionary conservation mapping of the Fsc1**.** Residues are colored according to conservation scores calculated by the ConSurf server, highlighting the distribution of highly conserved (burgundy) and variable (turquoise) residues across the FAS1 domains. Key structural features, including H1, H2, and the deep groove in FAS1-4, are indicated.
